# Patchouli Alcohol Protects the Heart against Diabetes-Related Cardiomyopathy through the JAK2/STAT3 Signaling Pathway

**DOI:** 10.3390/ph17050631

**Published:** 2024-05-14

**Authors:** Lijun Ji, Shuaijie Lou, Yi Fang, Xu Wang, Weiwei Zhu, Guang Liang, Kwangyoul Lee, Wu Luo, Zaishou Zhuang

**Affiliations:** 1 The Affiliated Cangnan Hospital and Chemical Biology Research Center, Wenzhou Medical University, Wenzhou 325000, China; jilijun968@gmail.com (L.J.); philou9882@hotmail.com (S.L.); f571467746@gmail.com (Y.F.); wangxu@ojlab.ac.cn (X.W.); 646675551y@gmail.com (W.Z.); liangguang@wmu.edu.cn (G.L.); 2 College of Pharmacy, Chonnam National University, Gwangju 61186, Republic of Korea; kwanglee@jnu.ac.kr; 3 School of Pharmaceutical Sciences, Hangzhou Medical College, Hangzhou 311399, China

**Keywords:** diabetic cardiomyopathy, patchouli alcohol, JAK2/STAT3 signaling pathway, inflammation, fibrosis

## Abstract

Diabetic cardiomyopathy (DCM) represents a common pathological state brought about by diabetes mellitus (DM). Patchouli alcohol (PatA) is known for its diverse advantageous effects, notably its anti-inflammatory properties and protective role against metabolic disorders. Despite this, the influence of PatA on DCM remains relatively unexplored. To explore the effect of PatA on diabetes-induced cardiac injury and dysfunction in mice, streptozotocin (STZ) was used to mimic type 1 diabetes in mice. Serological markers and echocardiography show that PatA treatment protects the heart against cardiomyopathy by controlling myocardial fibrosis but not by reducing hyperglycemia in diabetic mice. Discovery Studio 2017 software was used to perform reverse target screening of PatA, and we found that JAK2 may be a potential target of PatA. RNA-seq analysis of heart tissues revealed that PatA activity in the myocardium was primarily associated with the inflammatory fibrosis through the Janus tyrosine kinase 2 (JAK2)/signal transducer and activator of the transcription 3 (STAT3) pathway. In vitro, we also found that PatA alleviates high glucose (HG) + palmitic acid (PA)-induced fibrotic and inflammatory responses via inhibiting the JAK2/STAT3 signaling pathway in H9C2 cells. Our findings illustrate that PatA mitigates the effects of HG + PA- or STZ-induced cardiomyopathy by acting on the JAK2/STAT3 signaling pathway. These insights indicate that PatA could potentially serve as a therapeutic agent for DCM treatment.

## 1. Introduction

International Diabetes Federation reports that there were 537 million adults (20–79 years) with diabetes worldwide in 2021, and the number of adults with diabetes will increase to 783 million in 2045 [1]. In 1972, diabetic cardiomyopathy (DCM) was identified as cardiac dysfunction caused by diabetes in the absence of other cardiac risk factors [2,3]. Epidemiological studies shown that the prevalence of cardiac dysfunction with type 1 diabetes mellitus (T1DM, about 1 in 10 diabetes) and type 2 diabetes mellitus (T2DM, about 9 in 10) has been reported to be as high as 14.5% and 35.0%, respectively [4,5]. It is worth noting that the correlation between the glycated hemoglobin (HbA_1c_) level, a marker of glycemic control, and the risk of heart failure was significantly higher in patients with T1DM than those with T2DM [6]. However, achieving optimal glycemic control is still important to prevent and delay diabetes complications [7]. Unfortunately, multicenter randomized clinical studies have revealed that intensive glycemic control fails to reduce the mortality of patients with DCM especially in the advanced stage [8,9]. These studies suggested that transient hyperglycemia stress persists, a condition that is now referred to as ‘hyperglycemic memory’ (HGM) phenomenon [10]. It is very important to explore the mechanism of the HGM phenomenon and target this process to treat DCM.

Extensive research has scrutinized the multifaceted mechanisms underlying DCM. Contributory factors encompass inflammation, oxidative stress, aberrant immune responses, diminished bioavailability of nitric oxide, impaired autophagy in cardiomyocytes, and dysfunctional cardiac insulin metabolic signaling. Nitric oxide is a free radical that reacts with various molecules to cause multiple biological effects [11]. Nitric oxide also has cardioprotective effects, and nitric oxide signaling pathway has been proposed to link the adverse effects of persistent hyperglycemia with DCM [12]. These factors act synergistically to foster increased myocardial fibrosis, connective tissue cross-linking, and a pronounced elevation in cardiac stiffness, culminating in diastolic dysfunction [6,13]. Among these, inflammation serves as a pivotal player. Elevated concentrations of specific proinflammatory cytokines, namely, tumor necrosis factor-α (TNF-α) and interleukin-6 (IL-6), have been documented in patients with both DM and diastolic dysfunction [14,15]. Consequently, we advocate focusing on the inflammatory signaling pathway as a viable supplementary therapeutic approach for DCM in clinical settings. It is evident that mitigating these pathophysiological alterations is imperative for averting DCM and its related cardiovascular morbidities and mortalities [16]. Nonetheless, the scope of our understanding regarding how intensive management through various pharmacological agents impacts cardiovascular outcomes remains limited. Further research is imperative to elucidate the molecular underpinnings of DCM and to develop innovative preventive and therapeutic strategies.

JAK2 is a non-cross-model tyrosine kinase that is activated by tyrosine phosphorylation. The canonical JAK2/STAT3 pathway is a key mechanism for activating STAT3 transcription. Accumulating basic research using animal models has demonstrated the complex mechanisms and regulation of the JAK2/STAT3 pathway in pathological and physiological processes by mediating inflammatory and immune responses [17]. Cytokines can trigger the activation of the JAK2/STAT3 signaling pathway to modulate target gene expression [18]. However, the effects and associated mechanisms of the JAK2/STAT3 pathway on hyperglycemia-induced cardiomyocyte injury and inflammation remain unknown.

**Figure 1:**
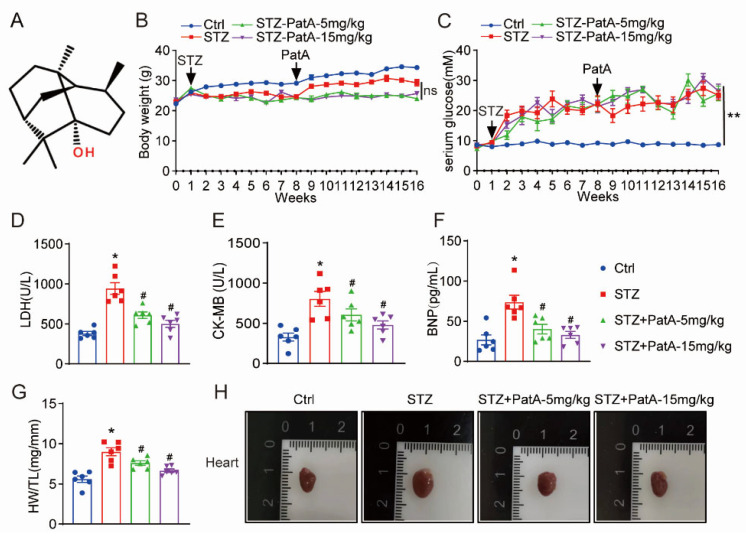
PatA ameliorates cardiac injury induced by diabetes. (**A**) Depicts the chemical structure of PatA. Mouse body weights (**B**) and fasting blood glucose levels (**C**) were tracked weekly over a period of 16 weeks. (**D**–**F**) Showcase the state of cardiac function and injury in mice, gauged through serum lactate dehydrogenase (LDH) (**D**), creatine kinase isoenzymes (CK-MB) (**E**), and brain natriuretic peptide (BNP) (**F**) at week 16. (**G**,**H**) Highlight the assessment of cardiac hypertrophy in mice, ascertained through the ratio of heart weight to tibia length (HW/TL) (**G**). (**H**) Provides illustrative images of hearts from different experimental groups (a sample size of 6 was used for each group; data are expressed as mean ± SEM; * *p* < 0.05, ** *p* < 0.01 relative to Ctrl; # *p* < 0.05 when compared with STZ; ns means not-significant).

*Patchouli alcohol* (PatA), a prominent bioactive component of the tricyclic sesquiterpene class (Figure 1A), is mainly extracted from parts of the *Pogostemon cablin* (*patchouli*) plant [19]. The literature of basic studies extensively documents the myriad of beneficial attributes of PatA, which include anti-influenza, anti-inflammatory, anti-ulcerogenic, and anti-colitis properties and protective effects on the lung, the brain, and the metabolic system [20]. Research underlines its anti-inflammatory efficacy in several disease models [21], notably, in conditions such as gastritis and brain injury induced by ischemia. In a recent study conducted by Wu et al., they provided evidence that PatA has the ability to alleviate intestinal mucositis caused by 5-fluorouracil, achieved by curbing the inflammatory signaling pathways mediated by the nuclear factor kappa B [22]. While the unique pharmacological attributes of PatA have attracted substantial scientific curiosity, a comprehensive understanding of the underlying mechanisms remains elusive. Furthermore, the exploration of PatA’s effects and fundamental mechanisms specifically in the context of DCM is yet to be undertaken.

Building on the background outlined above, this study aims to provide a comprehensive analysis of the scientific issues surrounding the effects of PatA on DCM, a disease caused by STZ-induced T1DM, and its mechanism of action. In our investigation, we explored the impact of PatA on inflammation and fibrosis of the myocardium using both in vitro and in vivo models. Under diabetic conditions, PatA effectively halted the amplification of proinflammatory molecules and excessive fibrosis, as demonstrated in both laboratory and animal studies. Further, we observed that PatA efficiently alleviated myocardial inflammation and fibrosis triggered by streptozotocin (STZ). This was achieved by hindering the phosphorylation process within the JAK2/STAT3 pathway. This particular action appeared to be closely associated with the preservation of cardiac function in the murine model.

## 2. Results

### 2.1. PatA Attenuates Diabetes-Induced Cardiac Injury and Dysfunction in Mice

In this experiment, diabetes was simulated in a murine model through the injection of STZ. Post the 8-week induction phase, the mice were administered two varying doses of PatA, specifically 5 and 15 mg/kg. Throughout this investigative period, weekly observations and recordings were made of body weight (BW) and blood glucose (BG) levels for all participating mice. As a notable outcome, the STZ-induced diabetic mice showcased a marked increase in the BG levels, in contrast to the control group, coupled with a decrease in body weight. Nevertheless, the administration of PatA appeared to have an insignificant impact on these observed levels (Figure 1B,C). Serum CK-MB, LDH, and BNP, which are known indicators of cardiac malfunction and injury, were subsequently evaluated. These indicators manifested an increased presence in the model group. Interestingly, PatA demonstrated a therapeutic effect, reducing the elevated levels of these markers, showing a reverse correlation with the administered drug quantity (Figure 1D–F). Diabetic cardiomyopathy (DCM) is often typified by cardiac hypertrophy, a condition where the heart muscle experiences an abnormal thickening, accompanied by an increase in heart tissue weight. A noteworthy rise in the heart weight/tibia length ratio was observed as a consequence of the STZ treatment. However, this impact was effectively mitigated by the administration of PatA (Figure 1G,H).

### 2.2. PatA Attenuates Diabetes-Induced Cardiac Injury and Myocardial Fibrosis in Mice

**Figure 2:**
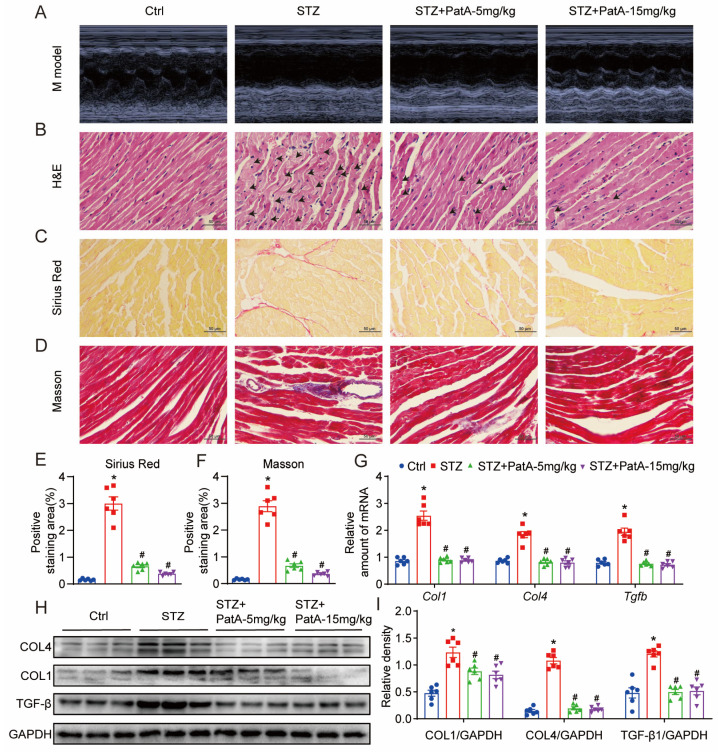
PatA alleviates myocardial fibrosis and cardiac damage induced by diabetes in mice. (**A**) Illustrates M-mode echocardiography images. (**B**) Displays images of heart tissues stained with H&E, and the arrows indicate myocardial structural irregularities. (**C**,**D**) Presents Sirius Red (**C**) and Masson trichromatic (**D**) staining images, and the collagen fibers in the tissue are stained red and blue-purple by Sirius red and Masson trichromatic staining, respectively. (**E**,**F**) Depicts Masson trichromatic (**E**) and Masson’s trichrome (**F**) staining in heart tissues and associated scores derived from quantification. (**G**) Shows RT-qPCR analysis results of Col1, Col4, and Tgfb mRNA levels in heart tissues. (**H**) Exhibits immunoblots indicating COL1, COL4, and TGF-β protein expressions in cardiac tissues. (**I**) Reflects the densitometric analysis of the blots shown in panel H. GAPDH was utilized as the loading control (each group contained 6 samples; data are represented as mean ± SEM; * *p* < 0.05 versus Ctrl; # *p* < 0.05 relative to STZ).

In an effort to evaluate the protective influence of PatA on diabetes-induced cardiac damage in murine models, echocardiographic analysis was carried out to determine cardiac performance. It was found that the diabetic mice exhibited a notable enlargement in left ventricular posterior wall thickness (LVPW) and left ventricular diameter (LVID), coupled with a downturn in EF% and FS% values (Table 1, Figure 2A). The adverse cardiac impacts prompted by diabetes were appreciably remedied by PatA, presenting a dose–response relationship. H&E staining provided supporting evidence that myocardial structural irregularities (marked with the arrows), a result of STZ induction, witnessed notable improvement post the PatA intervention (Figure 2B). Pathological remodeling in diabetes-provoked heart failure (HF) is often characterized by cardiac fibrosis. To affirm the anti-fibrotic influence of PatA in the cardiac tissues of diabetic mice, Sirius red staining and Masson’s trichrome staining were employed, targeting collagen (Figure 2C,E) and connective tissue (Figure 2D,F), respectively. Additionally, the instigation of cardiac fibrosis by STZ was corroborated through the elevated protein and mRNA expressions of COL4, COL1, and TGF-β1 in the heart, anomalies which were curtailed in a dose-dependent manner with PatA administration (Figure 2G–I). In summary, the protective function of PatA treatment on the heart against cardiomyopathy seems to predominantly stem from its regulatory effect on myocardial fibrosis, rather than mitigating hyperglycemia in the diabetic mice.

### 2.3. JAK2 May Be a Potential Target of PatA

**Figure 3:**
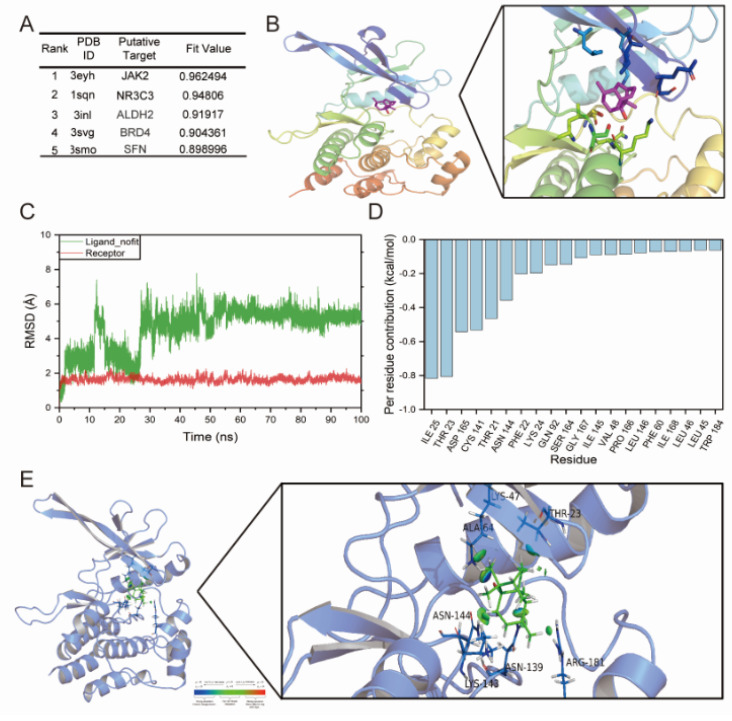
Prediction of targets and kinetic simulation for PatA. (**A**) The top five disease-related targets are ranked in the descending order based on their fit values, which represent the fraction of the ligand binding to the receptor. Higher fit values indicate high binding affinity. (**B**) Molecular docking of PatA with the JAK2 protein and the results of highest scoring molecular docking are presented. (**C**) A dynamic simulation was performed for a duration of 100 ns, and the resulting RMSD of small molecules is presented. (**D**) MM/GBSA calculations at the last 5 ns of the simulation trajectory were performed to derive the top 20 amino acid residues, which contributed to the free energy of small-molecule ligand binding to proteasome residues. (**E**) The visualization of the amino acid residues and the extent of interaction between JAK2 and PatA binding mode of small molecules, using the IGM mode.

To pinpoint the likely target of PatA, initial projections were carried out by employing the DS 2017 software. This allowed us to postulate the potential molecular targets for PatA and prioritize the top five based on descending fit values (as shown in Figure 3A). These fit values act as predictors of ligand–receptor binding, with higher scores implying superior binding. JAK2, a non-receptor tyrosine-protein kinase, is well recognized as a significant target for drugs used in treating inflammation or fibrosis [23,24]. In order to delve deeper into the binding association of PatA and JAK2, we undertook molecular docking and dynamics simulations. We started by docking PatA with the JAK2 protein and utilized the highest scoring outcome as the launch pad for the kinetic simulation (as seen in Figure 3B). Next, a molecular dynamics simulation lasting 100 nanoseconds (ns) was conducted. The results from this simulation showed that the RMSD of the small molecules fluctuated during the first 50 ns (Figure 3C) but achieved stability in the subsequent 50 ns period. In the closing 5 ns of the simulation trajectory, we calculated the free energy contribution of the 20 main amino acid residues to the small molecule ligand’s binding to proteasome residues (Figure 3D). This was achieved through the application of molecular mechanics coupled with generalized born and surface area solvation (MM/GBSA). Throughout the simulation, the protein-maintained stability. As depicted in the beginning and end stages of the simulation trajectory, the small molecule held its position within the protein’s pocket, with minuscule changes observed in its location. A further deep dive utilizing IGM unveiled the binding patterns of the small molecules (Figure 3E), illustrating the interactive amino acid residues and the degree of interaction.

### 2.4. PatA Alleviates DCM by Inhibiting the JAK2/STAT3 Pathway

**Figure 4:**
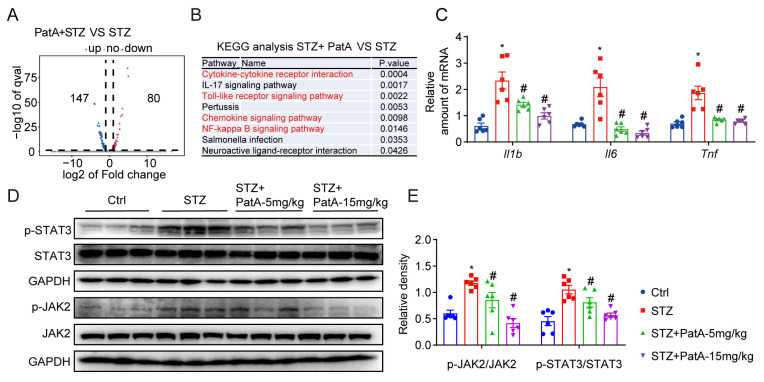
Illustration of how PatA mitigates the inflammatory response via JAK2/STAT3 pathway suppression. (**A**) A volcano plot highlights the alterations in heart tissue transcriptional patterns in diabetic mice subjected to PatA treatment versus untreated counterparts. Criteria were a fold change of >2 and a *p*-value of <0.01. Red and blue dots represent upregulated and downregulated genes, respectively, while gray dots signify genes without differential expression. (**B**) Kyoto Encyclopedia of Genes and Genomes (KEGG) pathway enrichment analysis shows significant term differences between groups (STZ vs. STZ + PatA). (**C**) Quantification of proinflammatory gene (Tnfa, Il1b, Il6) mRNA levels in the heart tissue of diabetic mice were performed via real-time quantitative PCR. (**D**) Heart tissue immunoblots demonstrate the proteins correlated with the JAK2/STAT3 pathway (JAK2, p-JAK2, STAT3, and p-STAT3). (**E**) Densitometric analysis of the blots from D was performed with GAPDH serving as the reference control. Each group comprised 6 samples; data are represented as mean ± SEM; significance is indicated by * *p* < 0.05 in comparison to Ctrl; # *p* < 0.05 relative to STZ.

To enhance our understanding of the cardioprotective mechanisms that PatA triggers, we utilized RNA sequencing analysis to study heart tissues from diabetic mice, both those treated and untreated with PatA. Our objective was to identify differentially expressed genes (DEGs). We discovered a total of 227 genes displaying variances in expression in myocardial tissues from diabetic mice who received PatA, when compared to those from the untreated group (as shown in Figure 4A). Insights gleaned from the KEGG pathway enrichment analysis indicated that the influence of PatA in the myocardium was primarily linked to the inflammatory response (as seen in Figure 4B). In support of this, RT-qPCR results corroborated that PatA treatment significantly subdued the upregulated proinflammatory genes Tnfa, Il1b, and Il6 in the cardiac tissues of diabetic mice (refer to Figure 4C).

Crucially, IL-6 is known to act as a common upstream activator of the JAK2/STAT3 pathway. To ascertain whether PatA impacts HG-induced inflammatory fibrosis via the JAK2/STAT3 pathway, we performed Western blot analysis. Our findings revealed that PatA substantially obstructed the activation of the JAK2/STAT3 pathway in heart tissues, demonstrating a dose-dependent effect (refer to Figure 4D,E).

### 2.5. PatA Alleviates HG + PA-Induced Fibrotic and Inflammatory Responses in Myocardial H9C2 Cells

**Figure 5:**
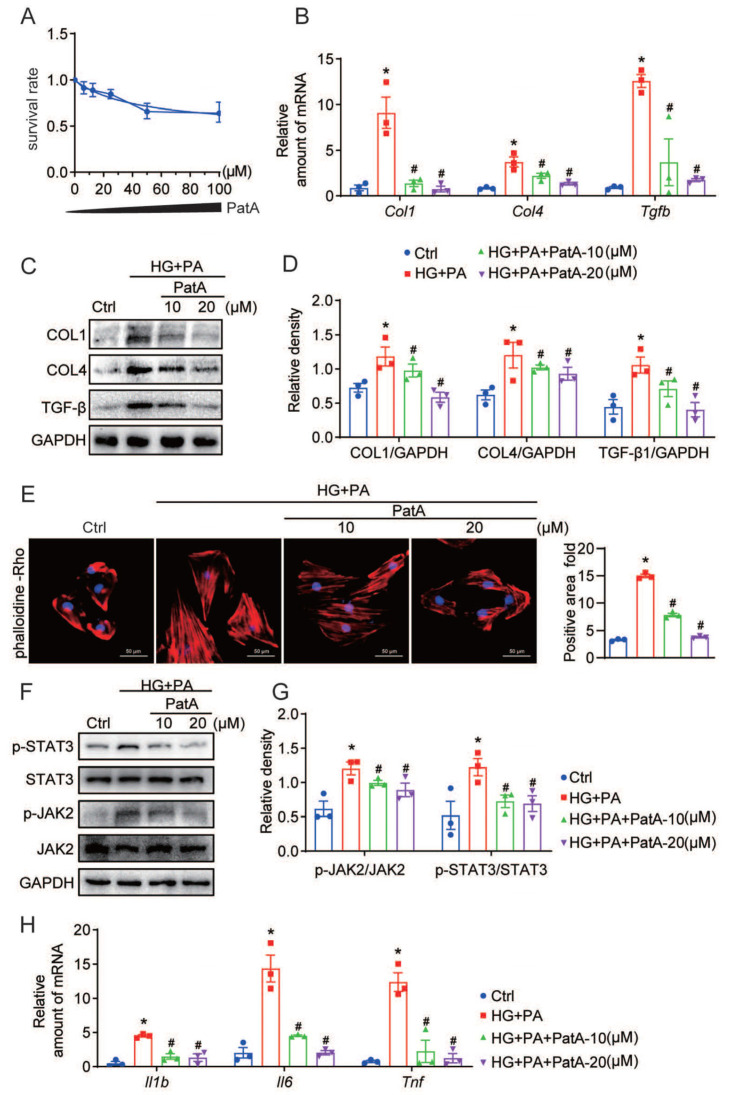
PatA’s role in diminishing HG-induced fibrogenic and inflammatory factors, and in blocking the activation of JAK2/STAT3 in H9C2 cells. (**A**) The cytotoxicity of varying concentrations of PatA (0–100 μM) on H9C2 cells was established via MTT (*n* = 3). (**B**–**D**) H9C2 cells underwent a pretreatment with 10 μM JAK2-IN for 1 h before being subjected to HG + PA (33 mM D-glucose + 200 μM PA) for an additional 24 h. (**B**) The transcript quantities of Col1, Col4, and Tgfb mRNAs were ascertained through real-time qPCR assay. (**C**) COL1, COL4, and TGF-β protein levels in H9C2 cells were evaluated through immunoblotting. (**D**) Densitometric analysis of immunoblots in C was performed, using GAPDH as the normalization control. (**E**) Phalloidin-rhodamine (red) staining of H9C2 cells illustrates the effect of PatA on hypertrophic reactions. The slides were further counterstained with DAPI (blue). (**F**) After being pretreated with 10 μM JAK2-IN for 1 h, H9C2 cells were exposed to HG + PA for 24 h. Subsequently, the proteins related to the JAK2/STAT3 pathway (JAK2, p-JAK2, STAT3, and p-STAT3) in H9C2 cells were revealed via immunoblots. (**G**) The densitometric analysis of the blots from F was conducted, using GAPDH as the reference control. (**H**) Following pretreatment with 10 μM JAK2-IN for 1 h, H9C2 cells were exposed to HG + PA for 24 h. mRNA levels of Il1b, Il6, and Tnf in H9C2 cells were measured by employing real-time quantitative PCR. Each group consisted of 3 samples; data are represented as mean ± SEM; * *p* < 0.05 indicates significant difference relative to Ctrl; # *p* < 0.05 demonstrates a significant difference compared to HG + PA.

In order to further assess the anti-inflammatory and anti-fibrotic molecular mechanisms elicited by PatA in DCM, we implemented in vitro experiments on H9C2 cells. The initial use of the MTT assay allowed us to determine PatA’s cytotoxicity in H9C2 cells (see Figure 5A). Utilizing the data derived from the MTT assay, we opted to focus on 10 and 20 μM concentrations to explore the subsequent impact of PatA. We discovered that the pre-administration of PatA effectively neutralized the HG + PA-provoked augmentations in Col4, Col1, and Tgfb mRNA levels (refer to Figure 5B). Utilizing Western blot techniques, we found that PatA worked in a dose-dependent manner to curb the protein levels of COL4, COL1, and TGF-β, which were otherwise elevated by HG + PA in H9C2 cells (as depicted in Figure 5C,D). Interestingly, PatA also reduced the hypertrophic cell response instigated by HG + PA, as signified by the results of RP staining (Figure 5E). Our in vitro findings resonated with our in vivo results, demonstrating that PatA treatment scaled back the HG + PA-induced phosphorylation of JAK2 and STAT3 in H9C2 cells, once again, in a dose-dependent manner (Figure 5F,G). RT-qPCR analysis further underscored the effects of PatA, showing a significant reduction in the HG + PA-induced overexpression of proinflammatory genes Tnfa, Il1b, and Il6 in H9C2 cells (Figure 5H). As predicted, PatA specifically obstructed the activation of JAK2 and STAT3 and lowered the inflammatory and fibrotic factors in HG + PA-stimulated H9C2 cells, thus demonstrating its potential cardioprotective attributes.

### 2.6. Inhibition of JAK2 Relieves HG + PA Induced-Myocardial Injury

**Figure 6:**
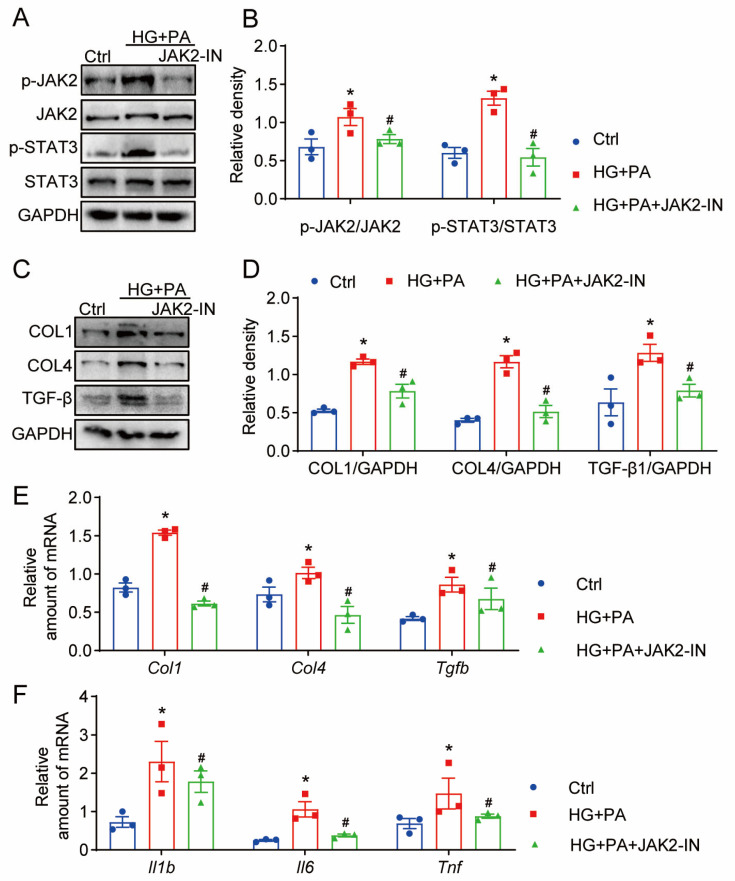
Mitigation of HG-induced fibrogenic and inflammatory factors in H9C2 through JAK2 inhibition (JAK2-IN). (**A**) H9C2 cells were subjected to pretreatment with 10 μM JAK2-IN for a duration of 1 h. Subsequently, the cells were exposed to a HG + PA combination (33 mM D-glucose +200 μM PA) for 24 h. Exhibited are representative Western blots demonstrating protein expression levels related to the JAK2/STAT3 pathway (JAK2, p-JAK2, STAT3, and p-STAT3). (**B**) Presents the densitometric analysis results of the blots from panel A, using GAPDH as the loading control. (**C**) After a 1 h pretreatment with 10 μM JAK2-IN, H9C2 cells were exposed to HG + PA for 24 h. Displayed are representative Western blots showing the expression levels of fibrosis-associated proteins (COL1, COL4, and TGF-β). (**D**) Densitometric analysis of the blots from panel C is provided, using GAPDH as the loading control. (**E**,**F**) H9C2 cells were pretreated with 10 μM JAK2-IN for 1 h and then exposed to HG + PA for 24 h. Quantified are (**E**) the mRNA levels of fibrosis-related proteins (Col1, Col4, and Tgfb) and (**F**) the proinflammatory cytokines (Il1b, Il6, and Tnfa), determined through real-time quantitative PCR (each group consisted of 3 samples; data are represented as mean ± SEM; * *p* < 0.05 versus Ctrl; # *p* < 0.05 relative to HG + PA).

To further scrutinize the function of the JAK2/STAT3 pathways in cardiomyocytes stimulated by HG + PA, we evaluated the impact of the JAK2 inhibitor on the inflammation and fibrosis of HG + PA-induced H9C2 cells. It became evident that the HG + PA group manifested an upsurge in the expression of p-JAK2 and p-STAT3. The JAK2 inhibitor proved effective in inhibiting JAK2/STAT3 phosphorylation in HG + PA-activated H9C2 cells (as depicted in Figure 6A,B). Through the application of Western blot analysis, we observed that the JAK2 inhibitor successfully reduced the expression of COL4, COL1, and TGF-β that were induced by HG + PA in H9C2 cells (Refer to Figure 6C,D). Furthermore, RT-qPCR assays signaled that HG + PA notably elevated the mRNA expressions of Col4, Col1, and Tgfb. However, this enhancement was attenuated by the preemptive administration of JAK2-IN in H9C2 cells (Figure 6E). Parallel findings were uncovered for proinflammatory genes, including Tnfa, Il1b, and Il1b (Refer to Figure 6F). Collectively, the outcomes suggest that the JAK2 inhibitor possesses the ability to suppress HG + PA-induced inflammation and fibrosis, acting through the JAK2/STAT3 pathway.

## 3. Discussion

Currently, the diagnosis, prevention, and therapy of DCM remain a great challenge due to its slow progression and atypical symptoms [2]. It is well established that hyperglycemia significantly contributes to the pathogenesis of DCM and irreversibly triggers a series of signal pathways that result in myocardial fibrosis and collagen deposition. Therefore, understanding the mechanisms that drive DCM progression and identifying potential therapeutic targets and drugs is paramount. Myocardial inflammation and fibrosis are two key pathophysiological mechanisms that drive cardiac remodeling and dysfunction resulting in heart failure [25]. Epidemiological studies suggest that hyperglycemia, a hallmark of either T1DM or T2DM, is closely associated with a chronic low-level inflammation that contributes to myocardial damage, leading to diabetic cardiomyopathy [26]. Given the central role of inflammation and fibrosis in the progress of diabetic cardiomyopathy, some anti-inflammatory drug-based and anti-fibrosis drug-based approaches have shown therapeutic effects on diabetic cardiomyopathy [27].

Natural compounds, including resveratrol, curcumin, troxerutin, and polyphenols, are renowned for addressing DCM through a variety of pathways [28]. In this investigation, we initially discovered that PatA significantly alleviated type 1 diabetes-induced cardiac damage and dysfunction in a dose-responsive manner. Often utilized for diagnosing cardiac dysfunction (such as left ventricular ejection fraction and time) in DCM is echocardiography [29]. Our findings highlighted the systolic and diastolic malfunctions induced by type 1 diabetes. The serum markers CK-MB, LDH, and BNP further verified the enhancement of cardiac function through PatA administration. Cardiac fibrosis, a signature of pathological remodeling in diabetes-induced heart failure (HF), was also addressed [30,31]. The structural anomalies and fibrosis brought on by diabetes were shown to be mitigated by PatA treatment via H&E, Sirius red, and Masson’s trichrome staining. TGF-β1, which is known to stimulate the growth of myocardial cells and blood vessels, particularly collagen synthesis, was found to be elevated in the myocardial tissue during DCM [32,33]. The diabetes-induced cardiac fibrosis was corroborated by increased COL4, COL1, and TGF-β1 protein and mRNA levels in the heart, which PatA successfully reversed.

Various network pharmacology applications were employed to forecast PatA’s potential targets, leading to the identification of several targets, including JAK2, a recognized upstream effector protein of STAT3. The JAK2/STAT3 pathway is a regulator of numerous cytokines that govern survival, proliferation, and differentiation of many cell types [34]. The continuous activation of the JAK2/STAT3 pathway fosters the advancement of a variety of diseases, encompassing myeloproliferative disorders, solid tumors, interstitial lung conditions, and osteoarthritis [35]. Conversely, numerous studies indicate that the activation of JAK2/STAT3 inhibits the onset of Alzheimer’s, Parkinson’s, and myocardial-cerebral vascular diseases [36,37]. Several observations have confirmed that high glucose has a major role in the upregulation of p-JAK2 and p-STAT3 in mesangial cells [38]. Mao et al. [39] found that the high-glucose-induced JAK/STAT signaling pathway is activated in human glomerular mesangial cells. The impact of the JAK2/STAT3 pathway in HG + PA-induced myocardial damage remains unclear. Several cytokines can incite the JAK2/STAT3 pathway, including interleukins, colony-stimulating factors, and interferons, all of which provoke an excessive inflammatory response under pathological conditions [40,41]. Our previous research has shown that STAT3 activation promotes the transcription of collagen-related genes, accelerating myocardial fibrosis in hypertension and DCM [42,43]. Marrero et al. [35] have noted that abnormal metabolites in diabetes patients, like angiotensin II and endothelin-1, can stimulate the activation of the JAK2/STAT3 pathway. Accordingly, obstructing this pathway has eased STZ-induced diabetic vascular complications in rats. Building on these insights, our findings further substantiate that the utilization of a specific JAK2 inhibitor (JAK2-IN) effectively suppresses HG + PA-induced inflammation and fibrosis through the inhibition of the JAK2/STAT3 pathway.

Moreover, our RNA-sequencing investigation pointed to a potential target pathway for PatA’s protective influence in DCM, specifically the cytokine-driven pro-inflammatory response pathway. This underscores the significance of focusing on this pathway to safeguard cardiac function in DCM. Subsequent verification using RT-qPCR tests revealed a substantial decrease in mRNA expression of Tnfa, Il1b, and Il6 post-PatA treatment, both in laboratory conditions and in living organisms. This evidence reinforces the premise that PatA delivers its favorable impact by diminishing inflammation within DCM. IL-6 is recognized as a traditional upstream instigator of the JAK2/STAT3 pathway [35]. We propose that the JAK2/STAT3 pathway is central to the cardioprotective effects of PatA in DCM. In line with our findings, a number of compounds (such as umbelliferone, phosphocreatine, taxifolin, and losartan) have been shown to mitigate DCM by suppressing the JAK2/STAT3 pathway [44,45].

## 4. Materials and Methods

### 4.1. Antibodies and Reagents

The compound PatA (Figure 1A; CAS#5986-55-0) was acquired from Alfa Biotechnology Co., Ltd., Chengdu, China. For in vitro applications, PatA was solubilized in dimethyl sulfoxide (DMSO). For in vivo studies, the compound was dissolved in a 1% solution of sodium carboxymethyl cellulose (CMC-Na). The JAK2 inhibitor, NVP-BSK805 (HY-14722A), was obtained from MedChemExpress Co., Ltd., based in Shanghai, China. Antibodies against JAK2 (Cat#3230) and STAT3 (Cat#9139) were sourced from Cell Signaling Technology, Danvers, MA, USA. Antibodies targeting collagen IV (COL4; Cat#Ab6586), collagen I (COL1; Cat#260043), transforming growth factor-β (TGF-β; Cat#Ab215715), and glyceraldehyde-3-phosphate dehydrogenase (GAPDH; Cat#Ab181602) were procured from Abcam, located in Cambridge, MA, USA. Antibodies specific to phospho-JAK2 (Tyr931) (Cat#AF3024) and phospho-STAT3 (Tyr705/Ser727) (Cat#AF5944) were supplied by Affinity Biosciences Co., Ltd., situated in Jiangsu, China. Staining kits for Hematoxylin and Eosin (H&E) (Cat#G1120), Sirius Red (Cat#G1472), and Masson’s Trichrome (Cat#G1340) were secured from Solarbio Science & Technology Co., Ltd., also based in Beijing, China.

### 4.2. Cell Culture

The terminally differentiated rat cardiac muscle cell line H9C2 [46,47] was sourced from the Shanghai Institute of Biochemistry and Cell Biology, Shanghai, China. These cells were cultured in Dulbecco’s Modified Eagle’s Medium (DMEM), which was provided by Gibco, Eggenstein, Germany. The medium was composed of 5.5 mmol/L D-glucose and was further supplemented with 10% fetal bovine serum (FBS). In addition, it was treated with 1 U/mL of penicillin and 1 mg/mL of streptomycin. To mimic diabetic conditions in vitro, the cells were exposed to the elevated concentrations of glucose (HG, 33 mmol/L) and palmitic acid (PA, 200 μmol/L).

### 4.3. Animals and Animal Models

Male mice of the C57BL/6 strain, with an age of eight weeks and weight range from 20 to 24 g, were sourced from the Beijing Vital River Laboratory Animal Technology Co., Ltd., located in Beijing, China. The Institutional Animal Care and Use Committee of Wenzhou Medical University granted official approval for all experimental procedures that involved these animals (approval No. wydw-2021-347). In terms of living conditions, the mice were maintained under a strictly adhered 12 h light–dark cycle and were fed with a routine rodent diet. We utilized these mice to establish a model of STZ-induced type 1 diabetes mellitus (T1DM), following protocols that have been previously standardized and validated in the field [48]. We employed STZ (Cat#S8050-1G; Sigma-Aldrich, St. Louis, MO, USA) as an effective diabetogenic agent. The STZ was carefully prepared in a 0.1 mol/L sodium citrate buffer (maintained at a pH of 4.5) and subsequently administered intraperitoneally at a dosage of 50 mg/kg/day over a period of five days. The control group of mice, which were non-diabetic (*n* = 6), were given solely the citrate buffer. Over the subsequent four weeks, blood glucose levels were measured on a weekly basis, using samples drawn from the mandibular vein and analyzed via a glucometer. The mice exhibiting fasting blood glucose levels beyond the threshold of 12 mmol/L were identified as diabetic.

To delve into the potential therapeutic effects of PatA, the mice were categorically divided into four distinct experimental groups (each comprising at least 6 mice): control (Ctrl), STZ-induced T1DM, STZ-induced T1DM mice administered with 5 mg/kg of PatA, and STZ-induced T1DM mice treated with 15 mg/kg of PatA [20,49]. Mice with diabetes were orally given PatA at two dose levels of 5 mg/kg (*n* = 6) and 15 mg/kg (*n* = 6) daily, while untreated diabetic control mice were administered the 1% CMC-Na vehicle (*n* = 6) instead. Throughout the duration of this 16-week study, we meticulously recorded both blood glucose levels and body weights on a weekly basis. Following the 16 weeks of PatA treatment, the mice were anesthetized through a 0.2 mL intraperitoneal injection of sodium pentobarbital at a concentration of 100 mg/mL, and subsequently euthanized to enable the collection of blood and heart tissue samples. Heart tissues were treated in two ways for further analysis, either they were snap-frozen using the optimal cutting temperature (OCT) technique or preserved in formalin for later paraffin embedding. We utilized specific assay kits (sourced from Nanjing Jiancheng, Nanjing, China) to quantify serum levels of lactate dehydrogenase (LDH), brain natriuretic peptide (BNP), and creatine kinase MB (CK-MB).

### 4.4. Echocardiography

Before their lives were humanely ended, the mice were sedated gently using 1.5% isoflurane. To ascertain the cardiac performance of these test subjects, we utilized the M-mode echocardiography technique, facilitated by a high-precision ultrasound imaging system specifically designed for small animals (Vevo 3100). More precisely, we captured M-mode and short-axis images as representative visual documentation. Through these images, we were able to calculate the ejection fraction (EF%) and fractional shortening (FS%), which are critical parameters for evaluating the cardiac systolic function. During the cyclical cardiac phases of diastole and systole, we took measurements of several vital parameters. These included the thickness of the left ventricular anterior wall during diastole (LVAWd) and systole (LVAWs), the thickness of the left ventricular posterior wall in diastole (LVPWd) and systole (LVPWs), and the left ventricular end-diastolic (LVEDV) and end-systolic volumes (LVESV). These data points served as quantitative indicators to assess the functionality of the myocardium in the test subjects.

### 4.5. Real-Time Quantitative PCR (RT-qPCR) Assay

We employed the use of Trizol Reagent (Cat#15596026; Thermo Fisher, Shanghai, China) for the extraction process of total RNA, with its concentration determined through the application of a SpectraMax M5 microplate reader (Molecular Devices, San Jose, CA, USA). To evaluate the purity of the extracted samples, we measured the optical density (OD) ratio (A260/A280), ensuring that it fell within the desired range of 1.8 to 2.2. Reverse transcription was performed using PrimeScriptTM RT Reagent Kit with gDNA Eraser (Cat. No. RR047A, Takara, Beijing, China). Quantitative PCR was performed using TB Green Premix Ex Taq^TM^ II (Cat. No. RR820A, Takara) in a QuantStudio^TM^ 3 Real-Time PCR System (Thermo Fisher Scientific). Relative expression was calculated by 2^ΔΔCt^ method with Actb normalization. Primer sequences are listed in Table 2. The primers were purchased from Tsingke Biotechnology Co., Ltd., Beijing, China.

### 4.6. Measurement of Biomarkers

The serum was obtained from isolated blood after centrifugation. Serum LDH, BNP, and CK-MB levels were measured using respective test kits (Nanjing Jiancheng, Nanjing, China), following the manufacturer’s instructions.

### 4.7. Histopathological Analysis

Following their collection, the heart specimens were accurately weighed. We then excised the apex and subsequently preserved it in a 4% paraformaldehyde solution. A gradual dehydration was carried out with an ascending alcohol gradient from 70% up to 100%. Ensuing this process, the tissue samples were embedded in paraffin, from which they were cut into thin sections. The next step involved rehydration of the samples, which were then stained using kits for H&E, Sirius red, and Masson’s trichrome, as directed by the supplier’s instructions. The objective here was to gain a clear understanding of the histopathological characteristics and determine the fibrosis stage. Capturing the images of the stained sections, we utilized an optical microscope (Nikon, Tokyo, Japan). The task of quantifying the representative images was undertaken using either the Image-Pro 10 or ImageJ software version 1.38e (National Institutes of Health, NIH, Bethesda, MD, USA). The digital image analysis system, Image-Pro (NIH, Bethesda, MD, USA), helped in quantifying the fibrotic region. A minimum of six non-overlapping fields were viewed, scored, and the derived values were related to the total tissue area in the field.

### 4.8. Western Blot Analysis

The initial processing of H9C2 cells or heart tissue involved homogenization with radioimmunoprecipitation assay (RIPA) lysis buffer (Cat#AR0103-100). We supplemented the lysis buffer with protease (Cat#HY-K0010) and phosphatase inhibitors (Cat#HY-K0021; Roche Applied Science, Indianapolis, IN, USA) to protect the proteins. The lysates underwent centrifugation at 12,000 rpm for a duration of 20 min. Subsequent to this, we extracted total proteins and estimated their concentration using the Bradford assay (BioRad Laboratories, Hercules, CA, USA). The preparation of protein loading samples was performed by blending the extracts with 5× SDS-PAGE protein loading buffer and water. Post the denaturation of proteins, they were separated on a 10% SDS-PAGE, with each lane accommodating 80 µg of protein. We then transferred the proteins electro-chemically to 0.45 µM polyvinylidene fluoride (PVDF) membranes (Bio-Rad, Hercules, CA, USA) using the wet transfer technique. The membranes were subsequently blocked at room temperature using a 5% skimmed milk solution in Tris-buffered saline with 0.05% Tween 20. After washing thrice with TBST, we incubated the membranes overnight at 4 °C with primary antibodies against various targets including p-JAK2 (1:1000), JAK2 (1:2000), p-STAT3 (1:1000), STAT3 (1:2000), COL4 (1:1000), COL1 (1:1000), TGF-β (1:1000), and GAPDH (1:1000). Following another three rounds of TBST washes, the membranes were incubated with the corresponding horseradish peroxidase-conjugated secondary antibodies at room temperature for 2 h. Post a final round of TBST washes, we conducted chemiluminescence detection on the membranes. The Molecular Imager VersaDoc MP 5000 system (Bio-Rad Laboratories, Inc., Hercules, CA, USA) was employed for band analysis, and the ECL reagent (NCM; Cat#P10300) was used for band exposure. Finally, we quantified the bands with the help of the Image J software, normalizing the values with respect to the loading control.

### 4.9. Methyl Thiazole Tetrazolium (MTT) Assay

We seeded H9C2 cells into 96-well plates, ensuring a density of 5000 cells within each well, and left them undisturbed to adhere overnight. Once the cells were successfully attached, we treated them with a range of PatA concentrations, which included 0, 3.125, 6.25, 12.5, 25, 50, 100, and 200 μM. This treatment was sustained for 24 h. Following the treatment period, we added the MTT reagent (Solarbio; Cat#M8180) into each well, and let it incubate for 4 h. After the incubation, we further added DMSO (Solarbio; Cat#D8371-50ML) into every well, which facilitated the dissolution of formazan crystals over a 10 min interval. We measured the absorbance in each well at a wavelength of 570 nm using a SpectraMax M5 microplate reader (Molecular Devices, San Jose, CA, USA). Finally, we represented the cell viability as a percentage in comparison to the control, providing a clear perspective on the effect of varying PatA concentrations on the H9C2 cells.

### 4.10. RNA Sequencing and Data Analysis

Collective heart specimens from each group were grouped into a single sample for RNA sequencing. Using a standardized approach, total RNA was harvested from the tissues. Following this, the total RNA’s purity and quantity were evaluated. Subsequently, a library was established and measured, with mRNA fragments serving as templates for cDNA generation. The yielded cDNAs were amplified using PCR to finalize the library. An effective concentration of the prepared library was then submitted for Illumina sequencing. To discern differentially expressed genes (DEGs), the normalization of the original read counts was carried out.

For the DEG assessment, the Kyoto Encyclopedia of Genes and Genomes (KEGG) pathway enrichment analysis was used. Differentially expressed mRNAs were handpicked based on the subsequent criteria: a fold change (FC) greater than 2 or less than 0.5, and a *p*-value lower than 0.05.

### 4.11. Molecular Docking and Dynamics Simulation

The assessment of the potential interaction between the investigational drug and the chosen protein was accomplished through reverse assays utilizing the Discovery Studio (DS) 2017 R2 software. Briefly, (1) the 3D structure of PatA (PDB Format) was constructed, and the structure was optimized and then submitted to the servers of Discovery Studio (DS) 2017 R2. (2) The ligand profiler was used to perform the reverse target screening of PatA based on the pharmacophore database. (3) The score of the fit value increased with stronger interactions between PatA and the corresponding protein. The protein database was procured from the Protein Data Bank’s collection (PharmaDB pharmacophores, New York, NY, USA). Within DS, ligands and pharmacophores were matched.

The bacterial JAK2 protein structure was derived from the RCSB Protein Data Bank (PDB code: 3eyh, New York, NY, USA). The potential binding regions within the receptor were identified. The ligand file was launched, and PatA’s potential binding to JAK2 was identified via LibDock molecular docking in the DS 2017 R2 software (Beijing, China). Default values were set as parameters. All of PatA’s postures were clustered using a tolerance of 2A for root-mean-square deviation (RMSD) and ranked based on the combined docking energy. For subsequent studies, the conformation with the lowest energy in the dense cluster was chosen. Nonbonded interactions between the receptor and ligand were analyzed, and a two-dimensional (2D) view showcased the ligand–receptor interactions.

The protein–ligand complex was imported into the computer interface to guarantee its activation. An isotropic displacement (also known as the B factor or the temperature factor) was applied to discern the level of uncertainty in each atom’s position. Following this, the protein was solvated, the force field was added, the standard dynamic cascade was opened, and the parameters were adjusted. The standard dynamic cascade process encompasses five stages: two optimization stages (I Minimization, I Minimization2), heating, equilibration, and simulation sampling (Production). The visualization of the results, alongside the display of the conformational change animation, allowed the 3D window complex to start moving, demonstrating the kinetic simulation changes. The ‘Analyze Trajectory’ method provided insights into the molecular dynamics trajectory’s structural information.

### 4.12. Rhodamine–Phalloidin (RP) Staining

In our quest to assess cellular hypertrophy, we adopted the RP staining method. In the initial phase, primary cardiomyocytes underwent fixation using a 4% paraformaldehyde solution, after which we permeabilized them with 0.1% Triton. Following this, we proceeded to stain these cells with phalloidin–rhodamine at a dilution ratio of 1:200. In the concluding steps of the procedure, we used 4′,6-diamidino-2-phenylindole (DAPI; Beyotime, Shanghai, China) for counterstaining. We meticulously examined the stained cells and captured their images using a Nikon 80i fluorescence microscope (Nikon, Tokyo, Japan), thus enabling us to closely observe and analyze cellular hypertrophy in these primary cardiomyocytes.

### 4.13. Statistical Analysis

Each experiment was conducted in a threefold manner. The applications SPSS 22.0 and GraphPad Prism 8 were utilized to conduct statistical analysis and create the graphs. Student’s *t*-test was used to analyze the differences between two groups, and one-way analysis of variance (ANOVA) was applied to determine the differences among multiple groups. A threshold of *p* < 0.05 was established as the indicator of statistical significance, and all statistical tests were two-tailed.

## 5. Limitation

T1DM and T2DM were usually induced by STZ only and a high fat diet (HFD) and STZ, respectively. Accordingly, HG and HG + PA treatments of cultured cells usually mimic T1DM and T2DM, respectively. However, our investigation primarily concentrated on the pharmacological effects of PatA in T1DM-induced myocardiopathy. We should explore the cardioprotective efficacy of PatA in T2DM-induced myocardiopathy in further research. This is an important limitation of our study. Some studies have reported that both the plasma glucose and free fatty acids were significantly increased in T1DM mice or rats [50,51], and some clinical studies also demonstrated that IR is a prominent feature of T1DM [52,53,54], and the HG + PA treatment of cultured cells was also used to mimic T1DM in our study. Unfortunately, there are not results for the lipid profile and the insulin sensitivity in T1DM mice. This is another important limitation of our study. Moreover, the direct interaction between PatA and JAK2 needs to be further verified by Surface Plasmon Resonance (SPR), drug affinity responsive target stability (DARTS) assay, or Cellular Thermal Shift Assay (CETSA). And the manner of PatA bind with JAK2 should be investigated by mutating the active amino acid sites predicted using the Discovery Studio (DS) 2017 R2 software.

## 6. Conclusions

In conclusion, our study elucidates that PatA ameliorates HG + PA- or STZ-induced cardiomyopathy through the modulation of the JAK2/STAT3 signaling pathway. Probing further into the mechanistic aspects, we found that PatA directly engages with JAK2, thereby attenuating the phosphorylation levels of JAK2/STAT3. These findings collectively suggest that PatA holds promise as a potential therapeutic candidate for the treatment of DCM. Our result show that PatA attenuates STZ-induced cardiac injury and dysfunction in mice independent of glycemic control. We speculate that PatA may be an alternative drug for the end stages of DCM and for patients with good glycemic control but experiencing DCM progression.

## Figures and Tables

**Table 1:** Echocardiographic analysis for cardiac injury and dysfunction in type 1 diabetic mice.

Parameters	Ctrl	STZ	STZ- PatA-5 mg/kg	STZ- PatA-15 mg/kg
	*n* = 6	*n* = 6	*n* = 6	*n* = 6
EF (%)	81.672 ± 0.046	76.483 ± 0.023 *	77.882 ± 0.025	80.263 ± 0.021 #
FS (%)	44.450 ± 0.052	39.121 ± 0.020 *	40.523 ± 0.023	42.753 ± 0.020 #
LVIDs (mm)	1.517 ± 0.231	1.583 ± 0.183 *	1.8 ± 0.126	1.683 ± 0.231 #
LVIDd (mm)	2.717 ± 0.183	2.6 ± 0.283 *	3.033 ± 0.288	2.933 ± 0.327 #
LVFWs (mm)	0.867 ± 0.052	0.783 ± 0.041 *	0.817 ± 0.075	0.8 ± 0.063 #
LVFWd (mm)	0.667 ± 0.082	0.6 ± 0.063 *	0.6 ± 0.063	0.633 ± 0.052 #
LVPWs (mm)	0.85 ± 0.055	0.8 ± 0 *	0.8 ± 0.063	0.833 ± 0.051 #
LVPWd (mm)	0.65 ± 0.055	0.6 ± 0.063 *	0.633 ± 0.051	0.617 ± 0.041 #

Data presented as mean ± SEM (*n* = 6; 1-way ANOVA followed by Tukey post hoc tests; number of comparisons, 10). Ctrl, control; EF, ejection fraction; FS, fractional shortening; LVIDd, diastole left ventricle internal dimension; LVFWd, diastole forward wall thickness; and LVPWd, diastole posterior wall thickness. * *p* < 0.05 compared with Ctrl. # *p* < 0.05 compared with STZ.

**Table 2:** Primer sequences for real-time qPCR assay.

Gene Name	Species	Primer Sequence (5′-3′)	
*Tgfb1*	Mouse	Forward	CTCCCGTGGCTTCTAGTGC
		Reversed	GCCTTAGTTTGGACAGGATCTG
*Co11a1*	Mouse	Forward	AATGGTGCTCCTGGTATTGC
		Reversed	GGTCCTCGTTTTCCTTCTT
*Co14*	Mouse	Forward	ATCGGATACTCCTTCCTCATGC
		Reversed	CCAGGGGAGACTAGGGACTG
*β-actin*	Mouse	Forward	GGCTGTATTCCCCTCCATCG
		Reversed	CCAGTTGGTAACAATGCCATGT
*Tnfa*	Mouse	Forward Reversed	CAGGGGCCACCACGCTCTTC TTTGTGAGTGTGAGGGTCTGG
*Il6*	Mouse	Forward Reversed	TAGTCCTTCCTACCCCAATTTCC TTGGTCCTTAGCCACTCCTTC
*Il1b*	Mouse	Forward Reversed	ACTCCTTAGTCCTCGGCCA CCATCAGAGGCAAGGAGGAA
*Col1a1*	Rat	Forward Reversed	GACATCCCTGAAGTCAGCTGC TCCCTTGGGTCCCTCGAC
*Tgfb1*	Rat	Forward Reversed	GCAACAACGCAATCTATGAC CCTGTATTCCGTCTCCTT
*Co14*	Rat	Forward Reversed	TAGGTGTCAGCAATTAGGCAGG TCACTTCAAGCATAGTGGTCCG
*β-actin*	Rat	Forward Reversed	TTCGCGTAGAAGAAGGCACAT CTTCCCATTCGTCAGTTTCCTC
*Tnfa*	Rat	Forward Reversed	GCCTCTTCTCATTTCCTGCTCGT CTCCGCTTGGTGGTTTGC
*Il6*	Rat	Forward Reversed	AGCCACTGCCTTCCCTACTTC AGTGCATCATCGCTGTTCATACAAT
*Il1b*	Rat	Forward Reversed	GCAACTGTTCCTGAACTCAACT ATCTTTTGGGGTCCGTCAACT

## Data Availability

The data presented in this study are contained within the article.

## Abbreviations

BNP	brain natriuretic peptide
BSA	bovine serum albumin
CK-MB	creatine kinase isoenzymes
CMC-Na	sodium carboxymethyl cellulose
COL1	collagen Type I
DCM	diabetic cardiomyopathy
DEGs	the differentially expressed genes
DMEM	Dulbecco’s Modified Eagle’s Medium
DMSO	dimethyl sulfoxide
ECL reagent	enhanced chemiluminescence reagent
FBS	fetal bovine serum
GAPDH	glyceraldehyde-3-phosphate dehydrogenase
H&E	hematoxylin and eosin
HG	high-concentration glucose
IL6	interleukin 6
JAK2-IN	JAK2 inhibitor
LDH	lactate dehydrogenase
MTT	methyl thiazolyl tetrazoliumlactate
OCT	optimal cutting temperature
PatA	patchouli alcohol
PVDF	polyvinylidene fluoride
qPCR	quantitative polymerase chain reaction
SDS-PAGE	sodium dodecyl sulfate polyacrylamide gel electrophoresis
STZ	streptozotocin
T1DM	type 1 diabetes mellitus
TBST	Tris-buffered saline with Tween-20
TGF-β	transforming growth factor beta
TL	tibia lengtht
TNF-α	tumor necrosis factor-α
